# Pretreatment Prognostic Factors for Intradiscal Condoliase Injection in Patients with Lumbar Disc Herniation: Insights from Clinical and MRI-Based Quantitative Analysis

**DOI:** 10.3390/jcm14051509

**Published:** 2025-02-24

**Authors:** Hideaki Nakajima, Arisa Kubota, Shuji Watanabe, Kazuya Honjoh, Naoto Takeura, Akihiko Matsumine

**Affiliations:** Department of Orthopaedics and Rehabilitation Medicine, University of Fukui Faculty of Medical Sciences, 23-3 Matsuoka Shimoaizuki, Eiheiji-cho, Yoshida-gun, Fukui 910-1193, Japan; akubota@u-fukui.ac.jp (A.K.); shujiw@u-fukui.ac.jp (S.W.); kazuya@u-fukui.ac.jp (K.H.); ntakeura@yahoo.co.jp (N.T.); mastumin@u-fukui.ac.jp (A.M.)

**Keywords:** lumbar disc herniation, intradiscal condoliase injection, pretreatment prognostic factor

## Abstract

**Background/Objectives**: Intradiscal condoliase injection is a minimally invasive and effective treatment option for lumbar disc herniation (LDH). However, the appropriate use, efficacy, and potential outcomes of this therapy have to be carefully considered because condoliase can only be administered once in life. The aim of this study is to identify factors that predict the efficacy of condoliase injection before treatment. **Methods**: A retrospective analysis was performed for 115 patients with LDH treated with intradiscal condoliase injection. The patients’ background and MRI-based evaluations were used to measure various pretreatment parameters, including age, sex, symptom duration, comorbidity of psychological factors, disc height, herniated mass area, and contrast ratios within the disc and herniation. Clinical response was defined as a ≥50% reduction in leg pain on a numerical rating scale 3 months after treatment. Factors with significance in the univariate analysis were further examined using multivariate logistic regression. **Results**: Among the 115 patients, 73.9% had a ≥50% post-treatment pain reduction. The predictive factors for poorer outcomes included a longer symptom duration, psychological comorbidities, a smaller herniated mass size, a higher disc height, and a higher contrast ratio within the disc. No significant associations were found between the treatment efficacy and patient age or contrast ratio within the herniation. **Conclusions**: This study identified several pre-treatment factors that predict the efficacy of intradiscal condoliase injection for LDH. Treatment decisions should be made with particular attention to patients with a longer symptom duration, psychological factors, and fewer degenerative discs. The most important determinant of treatment efficacy may be how condoliase acts on the herniation mass.

## 1. Introduction

Lumbar disc herniation (LDH) is a frequently encountered condition characterized by the compression of the nerve roots due to a degenerated nucleus pulposus. Conservative treatment is generally the first approach for patients with LDH; however, surgical intervention is necessary when conservative measures are ineffective. Common conservative therapies for LDH include oral medications, epidural blocks, and nerve root blocks. A randomized controlled trial (RCT) demonstrated that non-steroidal anti-inflammatory drugs were both better tolerated and more effective than placebo in the acute phase [1]. In contrast, a systematic review of 10 RCTs produced notably limited evidence, indicating that NSAIDs are as effective as placebos in reducing pain [2]. Regarding epidural corticosteroid injections, two systematic reviews found only short-term pain relief in patients with LDH when compared to the placebo [3,4]. A prospective study on therapeutic selective nerve block for LDH revealed that 90.7% of patients experienced immediate post-procedural pain relief; however, 61.8% later reported pain recurrence. The study indicated that surgery was avoided in up to 54% [5]. Chemonucleolysis using chondroitin sulfate ABC endolyase (condoliase) is an intermediate treatment between conservative and surgical interventions. The direct injection of condoliase into the intervertebral disc specifically degrades glycosaminoglycans, the main component of the nucleus pulposus, and reduces the water-holding capacity of proteoglycans.

Randomized controlled studies have shown that condoliase is both safe and efficacious for treating subligamentous extrusion-type LDH [6,7]. The drug regulatory authority in Japan granted approval for this treatment, and it became available in August 2018 as an intradiscal therapy for LDH. A meta-analysis using data from 10 studies (798 cases) gave a total effective rate of 78%, exceeding that of conservative treatment, with a surgery avoidance rate of 89% [8]. The use of intradiscal condoliase injection is increasing yearly [8], but since condoliase can only be administered once to avoid anaphylactic reactions, it is crucial for spine surgeons to understand its appropriate use, the prediction of its efficacy, and potential outcomes. Additionally, concerns such as the potential progression of intervertebral disc degeneration following treatment, the unverified efficacy of treatment in specific age groups (e.g., patients under 20 years or over 70 years of age), and a lack of clarity regarding prognostic factors in cases with limited efficacy further underscore the importance of identifying optimal candidates for this therapy.

More than 6 years after its launch, there have been many reports on the factors associated with the treatment efficacy of condoliase. Post-treatment intervertebral disc degeneration and a reduction in the herniated mass have been associated with an improved response [9,10]. The relationships between post-treatment changes and treatment effects have been widely examined, but there are few studies on the factors that predict outcomes before treatment. Pre-treatment disc degeneration and a high-intensity change in the herniated mass have been related to treatment outcomes, but this remains controversial. One possible reason for the inconsistent results in previous studies is the variability in the assessment of disc degeneration grades, such as that used in the Pfirrmann classification [11], and the presence of high-intensity changes within herniated discs, which may differ among observers. This highlights the need for more objective and reproducible metrics for evaluating magnetic resonance imaging (MRI) findings. Therefore, the aim of this study is to identify the factors that predict efficacy before treatment using the quantitative evaluation of imaging findings.

## 2. Materials and Methods

### 2.1. Study Population

Between May 2019 and May 2024, a total of 115 consecutive patients (average age 48.7 years; 81 males, 34 females) with LDH underwent intradiscal condoliase injection at our hospital. All had clinical indications of LDH. The eligibility criteria for intradiscal condoliase injection included unilateral lower extremity pain symptoms and ongoing neurological signs at the herniated disc level, as identified on high-resolution MRI. In all cases, these symptoms had been unresponsive to conservative treatments such as rest, medication, and nerve root block. Patients with motor and/or sensory disturbances, including bladder dysfunction and neurogenic intermittent claudication, as symptoms of lumbar spinal canal stenosis, were not indicated for intradiscal condoliase injection. Regarding LDH types, patients with multiple LDH, protrusion or sequestration-type LDH were excluded to ensure appropriate patient selection, whereas those with subligamentous extrusion or transligamentous extrusion types were included. Regarding disc degeneration, patients with Pfirrmann grade 5 discs were generally deemed less suitable for treatment due to advanced degeneration. However, a small number of patients with Pfirrmann grade 5 were included in this study. No patients in the study population had spinal instability. The study protocol was approved by Fukui University Medical Faculty Human Ethics Review Committee (Approval Number 2014046 and 20220210) and adhered strictly to the Japanese Government’s Ministry of Health, Labor, and Welfare Clinical Research Guidelines. The patients were informed that data from the research would be submitted for publication, and written informed consent was obtained from each patient.

### 2.2. Procedure

A board-certified spine surgeon performed the intradiscal injection of condoliase under fluoroscopic guidance with the patient in a semi-lateral position. The procedure involved the injection of a single 1 mL dose of condoliase (1.25 U/mL) into the center of the affected intervertebral nucleus pulposus. The injection was performed on the asymptomatic side using a 21-gauge puncture needle [6,7].

### 2.3. Data Collection and Clinical Assessments

Clinical and radiological assessments were conducted before and 3 months after the injection. A numerical rating scale (NRS) was used to evaluate the intensity of leg pain, with 0 representing no pain and 10 indicating the most severe pain ever experienced. The NRS before the treatment of the patients in this study was at least 5. Patients with a ≥50% reduction in leg discomfort three months post-injection, compared to their initial assessment, were categorized as responders. All others were classified as non-responders.

### 2.4. Patient Background

Data about the patients’ background, including their age, sex, the time from onset, prior discectomy at the same level, and psychological factors, were acquired from medical charts. Age was also classified into 4 groups (<20, 20–39, 40–70, and >70 years) and the association with treatment efficacy was assessed. The cutoff age limit was chosen based on previous reports [10,12] and package inserts. Psychological factors were defined as patients diagnosed with psychiatric disorders, including depression and somatic symptom disorders, by a neuropsychiatrist and/or taking medication at the time of intradiscal condoliase injection. These diagnoses were made based on established diagnostic criteria, such as those outlined in the Diagnostic and Statistical Manual of Mental Disorders (DSM)-5.

### 2.5. MRI Assessment

**Figure 1:**
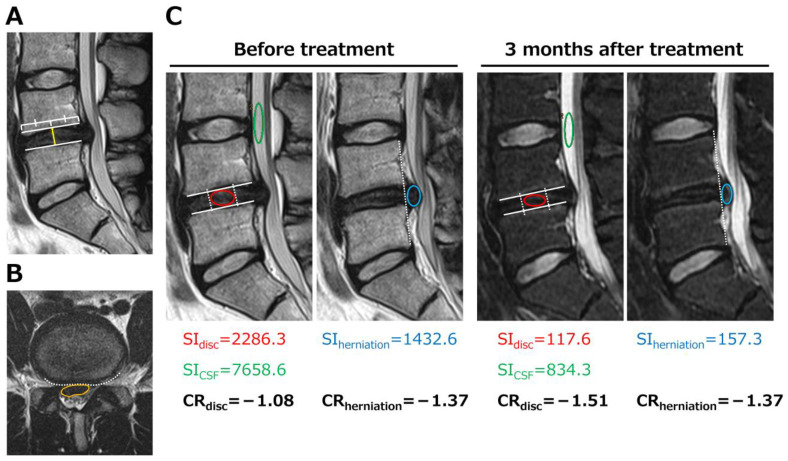
MRI findings. (**A**) Disc height at the midpoint of the vertebra (yellow line) on T2-weighted MRI. (**B**) The herniated mass area was quantified at the maximum bulging level on axial MRI (orange area), defined by reference lines drawn from the original dorsal surface of the annulus fibrosus (dotted line) to the dorsal surface of the herniated mass. (**C**) Images of the same patient before and after treatment. SI_disc_ was measured within the central 1/3 of the intervertebral disc (red area). SI_herniation_ was measured within the herniated region protruding beyond the posterior vertebral margin (dotted line) (blue area). SI_csf_ was obtained from the cerebrospinal fluid at the lumbar lesion level (green area). The contrast ratios (CR) were calculated as follows: CR_disc_ = (SI_disc_ − SI_csf_)/((SI_disc_ + SI_csf_)/2), and CR_herniation_ = (SI_herniation_ − SI_csf_)/((SI_herniation_ + SI_csf_)/2).

Axial and sagittal MRI were used to assess the presence of Modic type 1 changes in the vertebral bodies adjacent to the cartilage endplates at the affected disc. The herniated mass area, disc height, and signal intensity (SI) within the disc (SI_disc_) and herniation (SI_herniation_) were measured using a picture-archiving and communication system. Disc height (mm) was calculated at the midpoint of the vertebra on midsagittal MRI [9] (Figure 1A). The herniated mass area (mm^2^) at the maximum bulging level on axial MRI was measured by drawing reference lines enclosed by the original dorsal surface of the annulus fibrosus and the dorsal surface of the herniated mass (Figure 1B). SI values for the disc, herniation, and cerebrospinal fluid were obtained from T2-weighted sagittal MRI, with oval ROIs manually placed on the central 1/3 of the disc (SI_disc_) and bulged herniation mass (SI_herniation_). The SI values for the cerebrospinal fluid of the lumbar lesion were measured using oval ROIs (SI_csf_) (Figure 1C). The contrast ratios of the disc (CR_disc_) and herniation (CR_herniation_) were calculated using the following equation [13]: CR_disc_ = (SI_disc_ − SI_csf_)/((SI_disc_ + SI_csf_)/2) and CR_herniation_ = (SI_herniation_ − SI_csf_)/((SI_herniation_ + SI_csf_)/2). All measurements were performed in triplicate by two observers and average values were used.

### 2.6. Statistical Analysis

Data are presented as the mean ± standard deviation. Categorical variables were evaluated by the Mann–Whitney U-test or chi-square test, with *p* < 0.05 considered significant. Variables with significance in the univariate analysis were subsequently included in a multivariate logistic regression model. To determine the independent pre-treatment prognostic factors for intradiscal condoliase injection, odds ratios (OR) and corresponding 95% confidence intervals (CI) were calculated. The inter- and intraobserver reliability for radiological parameters was evaluated using intraclass correlation coefficients (ICCs), with ICC (2,3) >0.75 considered to indicate good to excellent reliability. Sample size calculations (*p* = 0.05, power = 0.80) and statistical power analysis were performed for each parameter. An analysis of the receiver operating characteristics (ROC) curve was applied to identify the best discriminating cut-off values for the pretreatment prognostic factors identified in this study. The optimal cut-off value was defined as the point on the curve closest to the upper left corner of the ROC graph, representing the best balance between sensitivity and specificity. Additionally, the Youden index was calculated to confirm the robustness of the identified thresholds. The area under the curve (AUC) was used to evaluate the accuracy of the parameter as a predictor. All analyses were conducted using EZR (Saitama Medical Center, Jichi Medical University, Saitama, Japan, Version 1.61) [14] and GUI for R (The R Foundation for Statistical Computing, Vienna, Austria).

## 3. Results

### 3.1. Demographic and Clinical Data

The demographic and clinical data of the enrolled patients are shown in Table 1. No outliers or anomalies were identified in the data during the analysis. Of the 115 patients, 85 (73.9%) were defined as responders (≥50% improvement in leg pain) and 30 (26.1%) were non-responders (<50% improvement). The mean age, age classification, and sex did not differ significantly between responders and non-responders at the time of injection. However, the time from onset to treatment was significantly longer in non-responders (41.4 vs. 24.4 weeks, *p* = 0.030, power = 0.86) and the proportion of patients with psychological factors was significantly higher in non-responders (26.7 vs. 7.1%, *p* = 0.012, power > 0.99). Of the seven patients with a history of discectomy at the same level, four (57.1%) were responders. Although this response rate appears to be low, there was no significant difference in efficacy between initial and revision cases (*p* = 0.55).

### 3.2. MRI Findings

The differences in MRI findings between responders and non-responders are shown in Table 2. The herniated mass area before treatment (*p* = 0.023, power = 0.80) and the % reduction in the herniated mass area after treatment (38.2% vs. 7.0%, *p* < 0.001, power > 0.99) were significantly higher in responders. The disc height before treatment was significantly higher in non-responders (*p* = 0.044, power = 0.61). The CR_disc_ before treatment was also significantly higher in non-responders (−1.05 vs. −1.19, *p* = 0.021, power = 0.81), whereas the CR_herniation_ before treatment was not associated with treatment efficacy. Changes in the disc height, changes in the CR_disc_, and the presence of a Modic type 1 change were not associated with treatment efficacy. The inter- and intraobserver reliabilities for the imaging findings were both excellent (*p* > 0.75).

### 3.3. Multivariate Analysis of Predictor of Treatment Efficacy

In multivariate logistic regression analysis, the time from onset (OR 0.98), comorbidity of psychological factors (OR 0.06), herniated mass area (OR 1.02), disc height (OR 0.68), and CR_disc_ (OR 0.77) before treatment were identified as independent and significant determinants of the treatment efficacy for intradiscal condoliase injection (Table 3). The AUC of the ROC curve for CR_disc_ before treatment was 0.62, and the cut-off for treatment efficacy was −0.77 (sensitivity, 95.2%; specificity, 23.3%). Of the 11 patients with −0.77 < pre-treatment CR_disc_ < 0, intradiscal condoliase injection showed a therapeutic effect in only 4 (36.4%). The cut-offs for the time from onset and disc height before treatment were 32.0 weeks (sensitivity, 85.0%; specificity, 40.0%; AUC, 0.66) and 9.5 mm (sensitivity, 82.1%; specificity, 40.0%; AUC, 0.61), respectively.

### 3.4. Representative Case

**Figure 2:**
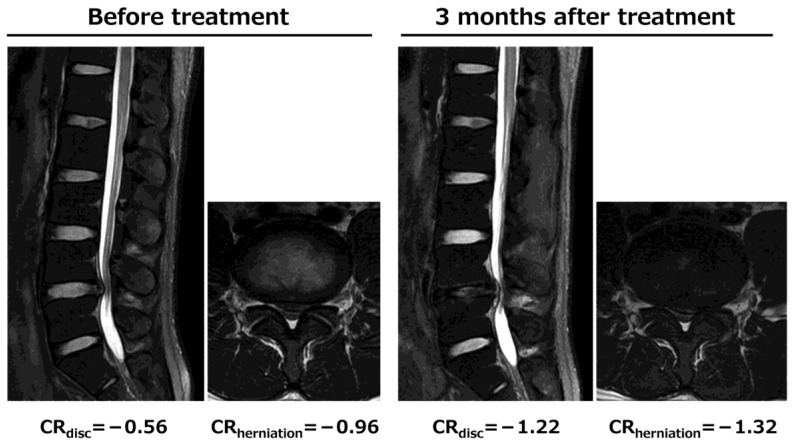
Representative non-responders with high disc height (10.6 mm) and high contrast ratio within the intervertebral disc (CR_disc_ = −0.56) before treatment. Only the progression of intervertebral disc degeneration without a reduction in the herniation mass volume was observed 3 months after treatment.

A representative case is shown in Figure 2. A 31-year-old male with L4-5 LDH experienced left lower extremity pain for 19 weeks. On MRI, the LDH (disc height of 10.6 mm, herniated mass area of 43.1 mm^2^, CR_disc_ of −0.56, CR_herniation_ of −0.96) was only slightly reduced 3 months after injection (disc height of 6.7 mm, herniated mass area of 36.2 mm^2^, CR_disc_ of −1.22, CR_herniation_ of −1.32, reduction rate of 16.0%). Only intervertebral disc degeneration progressed and the ligamentum flavum deflected, which progressed to spinal canal stenosis, and the NRS for leg pain changed from 8 at baseline to 9 at 3 months after treatment, indicating limited treatment efficacy.

## 4. Discussion

The aim of this study was to assess the pre-treatment prognostic factors influencing the therapeutic effects of intradiscal condoliase injection. The key predictors of the therapeutic effects of intradiscal condoliase injection before treatment were identified as follows: (1) a longer time from onset and comorbidity of psychological factors had a negative effect, (2) a larger herniated mass volume had a positive impact, and (3) a higher contrast ratio within the disc and a greater disc height (lower intervertebral disc degeneration with a remaining high-intensity area in the intervertebral disc) had a negative impact. The identified predictors have important implications for clinical decision-making regarding the use of intradiscal condoliase injections. For instance, patients with a symptom duration exceeding six months or those presenting with significant psychological comorbidities may benefit less from this treatment, suggesting that alternative therapeutic approaches should be considered in these cases. Similarly, patients with lower intervertebral disc degeneration and/or a greater disc height may not experience optimal outcomes, as the therapeutic agent may have limited penetration into the herniated region. These findings highlight the need for careful patient selection and suggest that the pretreatment evaluation of symptom duration, psychological factors, and imaging characteristics should guide treatment decisions.

Package inserts for condoliase indicate that the potential risks should be considered in different age groups. In younger patients (<20 years), there are concerns that spinal instability and cartilage ossification could be triggered due to impaired growth and development. In elderly patients (>70 years), warnings include the possibility of accelerated vertebral deterioration and reduced effectiveness resulting from the thinning of cartilage endplates and decreased proteoglycan levels. Chemonucleolysis with condoliase has been suggested to have limited efficacy in patients aged <20 years [15], but other studies have found that younger patients with LDH tend to have more significant symptom improvements following condoliase therapy [9,16], which may be due to an increased content of water in the nucleus pulposus in these patients. A significantly greater treatment efficacy has also been suggested in patients aged <40 years [10]. A meta-analysis of six studies (367 cases) found no significant difference in mean age between the responders and non-responders; there was also no correlation between age and treatment efficacy in the current study.

In this study, a longer duration of symptoms (41.4 vs. 24.4 weeks) was associated with reduced therapeutic efficacy. Previous studies have also indicated a much longer duration of symptoms among non-responders [9,17]. Other studies have found that non-responders have symptoms persisting beyond 12 months, but with no significant difference between responders and non-responders [18,19,20,21]. A randomized and observational cohort study suggested that a symptom duration >6 months was related to worse outcomes after conservative and surgical treatments [22]. Based on these findings, the symptom duration should not exceed 6 months.

The efficacy of intradiscal condoliase injection for recurrent LDH remains controversial. In a meta-analysis of five studies (31 cases), the odds ratio of the response rate was low (0.36) for recurrent cases compared to non-recurrent LDH, although the mean effective rate (72%) was as high as that for non-recurrent LDH (78%). A recent study suggested that intradiscal condoliase injection was less effective for recurrent LDH than primary LDH, with no significant improvement in lower back pain or ODI scores [23]. Although there was no significant difference, this finding suggests the need for the careful consideration of the indication of intradiscal condoliase injection for recurrent LDH.

In this study, the comorbidity of psychological factors was a strong independent negative predictor of the treatment efficacy of intradiscal condoliase injection, with an OR of 0.06. The narrow CI indicates a high degree of precision in the estimated effect size, reinforcing the clinical significance of this predictor. A recent study using the Hospital Anxiety and Depression Scale (HADS) also suggested that psychological factors, particularly depression, were significantly associated with a lower rate of effectiveness for intradiscal condoliase injection [15]. Patients with LDH may also have an increased risk of major depressive disorder compared to controls, even when accounting for demographic factors and coexisting health conditions [24]. Thus, for patients with LDH, psychological factors may be associated with the underlying mechanisms and degree of pain experienced.

Package inserts state that the indication for intradiscal condoliase injection is limited to subligamentous extrusion-type LDH, but there are scattered reports of an equivalent efficacy for transligamentous extrusion-type LDH [9,10,17,19,25]. On the other hand, a systematic review and meta-analysis suggested that a larger initial herniation size and the absence of Modic changes are associated with spontaneous regression in LDH: 70.4% in extruded LDH and 93.0% in sequestered-LDH [26]. When considering the use of intradiscal condoliase injection, particularly in cases of transligamentous extrusion-type LDH, spine surgeons should be wary of implementing therapy too early, given the potential for spontaneous regression. Modic changes in LDH cases may be associated with cartilaginous herniations and less neovascularization and macrophage infiltration, resulting in less spontaneous regression [27]. In this study, the presence of Modic type 1 changes before treatment was not associated with the efficacy of intradiscal condoliase injection.

Recent research indicates that the progression of disc degeneration following treatment may be a positive indicator of treatment effectiveness, despite the lack of correlation between pre-treatment disc degeneration levels and response rates [10]. The association between MRI findings with a high-intensity area in LDH and a good treatment efficacy have been described [9,19,21,28], but the results remain controversial. The classification of the degree of disc degeneration and its evaluation based on the presence of a high-intensity area in LDH may be ambiguous due to the low inter- and intraobserver reliability. In radiological analysis, CR is used as a quantitative parameter for evaluating human tissue composition [13,29,30]. In the present study, to standardize SI, cerebrospinal fluid was used as a reference, since this varies minimally among individuals, and we refer to this standardized measure as CR. The evaluation of CR is advantageous because of the relative ease of measurement using standard MRI, regardless of the imaging method or model. Based on our quantitative analysis, CR_herniation_ was not associated with the treatment outcome, whereas a higher CR_disc_, reflecting relatively preserved intradiscal signal intensity, was associated with poorer therapeutic efficacy (OR 0.77). The results suggested that −0.77 < CR_disc_ < 0 is an important indicator of poor treatment efficacy. However, because the cut-off specificity was low and wide CI, CR_disc_ < −0.77 was not indicative of high treatment efficacy. The degree of disc degeneration before treatment has been suggested not to affect treatment efficacy, but our results suggest that the indication should be more carefully evaluated in cases with disc hyperintensity changes above a certain level and/or a greater disc height.

**Figure 3:**
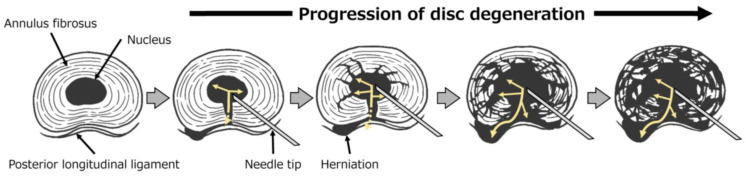
Schematic of disc degeneration. Intervertebral disc degeneration begins with a fissure in the center of the posterior annulus fibrosus, followed by the progressive disruption of the annulus fibrosus. Arrows: predicted condoliase spread in the disc; dotted arrows: difficulty with condoliase spread.

The process of disc degeneration based on discography studies [31] is shown in Figure 3. Intervertebral disc degeneration is thought to begin with a fissure in the center of the posterior annulus fibrosus, followed by the progressive disruption of the annulus fibrosus as the disc degenerates. The key determinant of treatment success may be how effectively condoliase acts and spreads within the disc and into a herniated region. In patients with low disc degeneration and/or a high disc height, it may be difficult for condoliase to reach the herniation or it may spill over into the intervertebral disc, causing disc degeneration and/or decreasing the disc height without reducing the herniation size. In such cases, intradiscal condoliase injection is unlikely to be effective. This may explain why CR_herniation_ was not associated with treatment outcomes in this study.

This study has several limitations, including its retrospective, single-center design, the small number of patients for statistical analysis, and the lack of a control group for comparison with patients receiving conservative management or surgical intervention. Although the inter- and intraobserver reliability for the manual measurement of MRI parameters was high in this study, the process of manually setting ROIs may introduce some degree of subjectivity and variability. Larger-scale population studies are needed to provide further evidence to validate our results. Specifically, future prospective trials should focus on incorporating a control group and standardizing measurement protocols to minimize variability. In addition, validating the identified MRI and clinical predictors as biomarkers for treatment efficacy will be essential to establish their clinical utility. Advanced imaging techniques and machine learning approaches could also be explored to refine the prediction models and improve the accuracy of patient selection for condoliase therapy. Despite these limitations, we believe that the findings provide important insights and guidance regarding the indication for intradiscal condoliase injection in patients with LDH through the identification of pre-treatment predictors of efficacy.

## 5. Conclusions

Our findings identified several key pre-treatment prognostic factors that can inform clinical decision-making regarding the use of intradiscal condoliase injection. Specifically, a longer time from onset, psychological factor comorbidities, a smaller herniated mass volume, a higher contrast ratio within the disc (lower intervertebral disc degeneration with a remaining high-intensity area), and a greater disc height before treatment were negative prognostic factors for the treatment efficacy of intradiscal condoliase injection. These results highlight the importance of careful patient selection based on these predictors to optimize therapeutic outcomes.

## Figures and Tables

**Table 1:** Comparison of clinical data at baseline for responders and non-responders.

Parameter	Responders	Non-Responders	*p* Value
Case number, *n* (%)	85 (73.9%)	30 (26.1%)	
Age			
Average (years)	49.1 ± 17.5	47.4 ± 17.3	0.66
Classification, *n* (%)			0.58
<20	2 (2.4%)	0 (0%)	
20–39	23 (27.1%)	8 (26.7%)	
40–70	45 (52.9%)	19 (63.3%)	
>70	15 (17.6%)	3 (10.0%)	
Sex, *n*	Male 58, Female 27	Male 23, Female 7	0.52
Time from onset (weeks)	24.4 ± 29.9	41.4 ± 37.8	0.030 *
Prior discectomy at the same level, *n* (%)	4 (4.7%)	3 (10.0%)	0.55
Psychological factor, n (%)	6 (7.1%)	8 (26.7%)	0.012 *

* *p* < 0.05.

**Table 2:** Comparison of MRI findings for responders and non-responders.

Parameter	Responders (*n* = 85)	Non-Responders (*n* = 30)	*p* Value
**Pre-treatment factors**			
Herniated mass area (mm^2^)	90.9 ± 36.3	73.7 ± 32.5	0.023 *
Disc height (mm)	8.3 ± 1.8	9.0 ± 1.7	0.044 *
Contrast ratio			
within disc	−1.19 ± 0.25	−1.05 ± 0.33	0.021 *
within herniation	−1.32 ± 0.26	−1.34 ± 0.31	0.71
Pfirrmann grade			0.28
grade 2 and 3	48 (3–45)	21 (9–12)	
grade 4 and 5	37 (36–1)	9 (7–2)	
Modic type 1 change, n (%)	9 (10.6%)	4 (13.3%)	0.94
**Post-treatment factors**			
Reduction rate of herniated mass area (%)	38.2 ± 26.5	7.0 ± 30.8	<0.001 *
Change in disc height (mm)	1.4 ± 0.9	1.7 ± 1.5	0.24
Change in contrast ratio within disc	−0.15 ± 0.27	−0.12 ± 0.23	0.68
Modic type 1 change, n (%)	24 (28.2%)	9 (30.0%)	<0.99

* *p* < 0.05.

**Table 3:** Multivariate logistic regression analysis of pre-treatment prognostic factors.

Variables	Odds Ratio	95% CI	*p* Value
Time from onset (per 1 week)	0.98	0.96–1.00	0.019
Psychological factor (Yes vs. No)	0.06	0.01–0.32	0.0011
Herniated mass area (per 1 mm^2^)	1.02	1.00–1.04	0.022
Disc height (per 1 mm)	0.68	0.47–0.98	0.039
Contrast ratio within intervertebral disc (per 0.1 increase)	0.77	0.61–0.97	0.030

## Data Availability

The data presented in this study are available upon request from the corresponding author and subject to the ethical approvals in place and material transfer agreements.

## Abbreviations

The following abbreviations are used in this manuscript:

LDH	Lumbar disc herniation
MRI	Magnetic resonance imaging
NRS	Numerical rating scale
SI	Signal intensity
CR	Contrast ratio
OR	Odds ratio
CI	Confidence intervals
ICC	Intraclass correlation coefficients
ROC	Receiver operating characteristic
AUC	Area under the curve
HADS	Hospital Anxiety and Depression Scale
