# Correction: Nakajima et al. Pretreatment Prognostic Factors for Intradiscal Condoliase Injection in Patients with Lumbar Disc Herniation: Insights from Clinical and MRI-Based Quantitative Analysis. *J. Clin. Med.* 2025, *14*, 1509

**DOI:** 10.3390/jcm15103760

**Published:** 2026-05-14

**Authors:** Hideaki Nakajima, Arisa Kubota, Shuji Watanabe, Kazuya Honjoh, Naoto Takeura, Akihiko Matsumine

**Affiliations:** Department of Orthopaedics and Rehabilitation Medicine, University of Fukui Faculty of Medical Sciences, 23-3 Matsuoka Shimoaizuki, Eiheiji-cho, Yoshida-gun, Fukui 910-1193, Japan; akubota@u-fukui.ac.jp (A.K.); shujiw@u-fukui.ac.jp (S.W.); kazuya@u-fukui.ac.jp (K.H.); ntakeura@yahoo.co.jp (N.T.); mastumin@u-fukui.ac.jp (A.M.)

## Error in Table

In the original publication [1], there was a mistake in Table 3. There was an error in the calculation of CRdisc used in the multivariate logistic regression analysis shown in Table 3. During data preparation, the formula for CRdisc was inadvertently entered in reverse order, resulting in inversion of the sign of CRdisc values in the multivariate model. Consequently, the odds ratio, confidence interval, and related text required correction. The univariate analyses and all other variables were unaffected. After reanalysis using the correct formula, the statistical significance and scientific conclusions remain unchanged. The corrected Table 3 appears below. 

## Text Correction

A correction has been made to Section 3.3. Multivariate Analysis of Predictor of Treatment Efficacy and Section 4. Discussion, paragraph 7.

3.3. Multivariate Analysis of Predictor of Treatment Efficacy

In multivariate logistic regression analysis, the time from onset (OR 0.98), comorbidity of psychological factors (OR 0.06), herniated mass area (OR 1.02), disc height (OR 0.68), and CR_disc_ (OR 0.77) before treatment were identified as independent and significant determinants of the treatment efficacy for intradiscal condoliase injection (Table 3). The AUC of the ROC curve for CR_disc_ before treatment was 0.62, and the cut-off for treatment efficacy was −0.77 (sensitivity, 95.2%; specificity, 23.3%). Of the 11 patients with −0.77 < pre-treatment CR_disc_ < 0, intradiscal condoliase injection showed a therapeutic effect in only 4 (36.4%). The cut-offs for the time from onset and disc height before treatment were 32.0 weeks (sensitivity, 85.0%; specificity, 40.0%; AUC, 0.66) and 9.5 mm (sensitivity, 82.1%; specificity, 40.0%; AUC, 0.61), respectively.

4. Discussion, Paragraph 7

Based on our quantitative analysis, CR_herniation_ was not associated with the treatment outcome, whereas a higher CR_disc_, reflecting relatively preserved intradiscal signal intensity, was associated with poorer therapeutic efficacy (OR 0.77).

The authors state that the scientific conclusions are unaffected. This correction was approved by the Academic Editor. The original publication has also been updated.

## Figures and Tables

**Table 3:** Multivariate logistic regression analysis of pre-treatment prognostic factors.

Variables	Odds Ratio	95% CI	*p* Value
Time from onset (per 1 week)	0.98	0.96–1.00	0.019
Psychological factor (Yes vs. No)	0.06	0.01–0.32	0.0011
Herniated mass area (per 1 mm^2^)	1.02	1.00–1.04	0.022
Disc height (per 1 mm)	0.68	0.47–0.98	0.039
Contrast ratio within intervertebral disc (per 0.1 increase)	0.77	0.61–0.97	0.030

## References

[1] Nakajima H., Kubota A., Watanabe S., Honjoh K., Takeura N., Matsumine A. (2025). Pretreatment Prognostic Factors for Intradiscal Condoliase Injection in Patients with Lumbar Disc Herniation: Insights from Clinical and MRI-Based Quantitative Analysis. J. Clin. Med..

